# Design, Synthesis and Biological Evaluation of 5-Oxo-1,4,5,6,7,8 Hexahydroquinoline Derivatives as Selective Cyclooxygenase-2 Inhibitors 

**Published:** 2014

**Authors:** Afshin Zarghi, Iman Sabakhi, Vigen Topuzyan, Zahra Hajimahdi, Bahram Daraie

**Affiliations:** a Department of Medicinal Chemistry, School of Pharmacy, Shahid Beheshti University of Medical Sciences, Tehran, Iran.; b The Scientific Thecnological Centre of Organic and Pharmaceutical Chemistry NASRAAL. Mnjoyan Institute of Fine Organic Chemistry, Yerevan, Armenia.; c The Scientific Thecnological Centre of Organic and Pharmaceutical Chemistry NASRAAL. Mnjoyan Institute of Fine Organic Chemistry, Yerevan, Armenia.; d Department of Pharmaceutical Chemistry, School of Pharmacy, Shahid Beheshti University of Medical Sciences, Tehran, Iran.; e Department of Toxicology, School of Medicine, Tarbiat Modarres University of Medical Sciences, Tehran, Iran.

**Keywords:** 5-Oxo-1, 4, 5, 6, 7, 8 hexahydroquinolines, COX-2 Inhibitors, Molecular modeling, Hansch condensation

## Abstract

A group of regioisomeric 5-oxo-1,4,5,6,7,8 hexahydroquinoline derivatives possessing a COX-2 SO2Me pharmacophore at the para position of the C-2 or C-4 phenyl ring, in conjunction with a C-4 or C-2 phenyl (4-H) or substituted-phenyl ring (4-F,4-Cl,4-Br,4-OMe,4-Me, 4-NO_2_), were designed for evaluation as selective cyclooxygenase-2 (COX-2) inhibitors. These target 5-oxo-1,4,5,6,7,8 hexahydroquinolines were synthesized via a Hansch condensation reaction. In vitro COX-1/COX-2 isozyme inhibition structure-activity studies identified 7,8-dihydro- 7,7-dimethyl-2-(4-methoxyphenyl)-4-(4-(methylsulfonyl)phenyl)quinolin-5(1H,4H,6H)- one (9c) as a potent COX-2 inhibitor (IC50 = 0.17 M) with a high COX-2 selectivity index (S.I. = 97.6) comparable to the reference drug celecoxib (COX-2 IC50 = 0.05 mM; COX-2 S.I= 405). A molecular modeling study where 9c was docked in active site of COX-2 showed that the p-SO2Me substituent on the C-2 phenyl ring is inserted into the secondary COX-2 binding site. The structure activity data acquired indicate that the position of the COX-2 SO2Me pharmacophore and type of substituent are important for COX-2 inhibitory activity.

## Introduction

**Figure 1:**
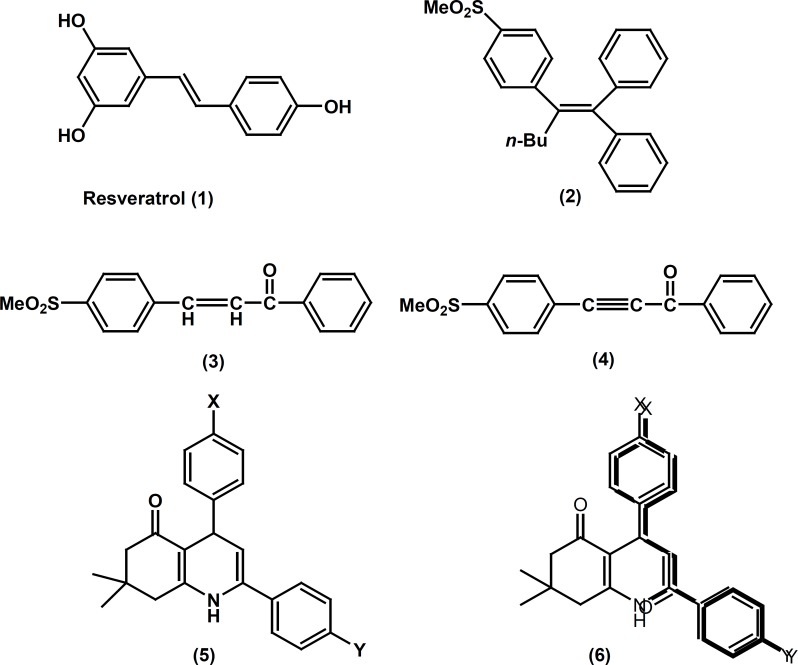
Some representative examples of a selective cyclooxygenase-1 (1), cyclooxygenase-2 (2-4) inhibitors, designed molecules (5) and overlay of our design molecules on lead compound 3 (6).

Selective cyclooxygenase-2 (COX-2) inhibitors frequently belong to a class of diarylheterocycles that possess two vicinal rings attached to a central heterocyclic scaffold in conjunction with a COX-2 pharmacophore such as a *para*-SO2Me substituent on one of the rings (1). Compounds having an acyclic central scaffold have also been identified that exhibit COX inhibitory activity. Accordingly, resveratrol (1) possessing *trans*-olefin system displays COX-1 selectivity (2). In contrast, it showed that the 1,1.2-tiraryl (*Z*)-olefin (2) (3), the 1,3-diphenylprop-2-en-1-one (3) (4) and the 1,3-diphenylprop-2-yn-1-one (4) (5) exhibit not only potent, but also highly selective, COX-2 inhibitory activity (see structures 1-4 in Figure 1). Recently, we reported several investigations describing the design, synthesis, and anti-inflammatory properties for a novel class of compounds possessing an acyclic 1, 3-diphenylprop-2-en-1-one structural template. Our results showed that the propenone moiety is a suitable scaffold (template) to design COX-2 inhibitors (4, 6, 7). As part of our ongoing program to design new types of selective COX-2 inhibitors, we now report the synthesis, some structure-activity relationships, and a molecular modeling study for a group of 5-oxo-1,4,5,6,7,8 hexahydroquinoline regioisomers possessing a COX-2 SO2Me pharmacophore at the *para*-position of one phenyl ring in conjunction with a substituent (4-F, 4-Cl, 4-Br, 4-OMe, 4-Me, 4-NO2) at the *para*-position of the other phenyl ring. In this study we utilized the 1, 3-diphenylprop-2-en-1-one moieties as a part of our designed molecules.

## Experimental

*General*

All chemicals and solvents used in this study were purchased from Merck AG and Aldrich Chemical. Melting points were determined using a Thomas-Hoover capillary apparatus. Infrared spectra were acquired using a Perkin Elmer Model 550 SE spectrometer. A Bruker AM-300 NMR spectrometer was used to acquire 1H NMR spectra with TMS as internal standard. Coupling constant (*J*) values are estimated in hertz (Hz) and spin multiples are given as s (singlet), d (double), t (triplet), q (quartet), m (multiplet), and br (broad). Low-resolution mass spectra were acquired with an MAT CH5/DF (Finnigan) mass spectrometer that was coupled on line to a Data General DS 50 data system. Electron-impact ionization was performed at an ionizing energy of 70 eV with a source temperature of 250 oC. Elemental microanalyses, determined for C and H, were within ±0.4% of theoretical values. All chemicals and solvents used in this study were purchased from Merck AG and Aldrich Chemical. Melting points were determined with a Thomas– Hoover capillary apparatus. Infrared spectra were acquired using a Perkin Elmer Model 1420 spectrometer. A Bruker FT-500 MHz instrument (Bruker Biosciences, USA) was used to acquire 1HNMR spectra with TMS as internal standard. Chloroform-D was used as solvents. Coupling constant (*J*) values are estimated in hertz (Hz) and spin multiples are given as s (singlet), d (double), t (triplet), q (quartet), m (multiplet) and br (broad). The mass spectral measurements were performed on a 6410 Agilent LCMS triple quadrupole mass spectrometer (LCMS) with an electrospray ionization (ESI) interface. 

*Chemistry*

**Scheme 1:**
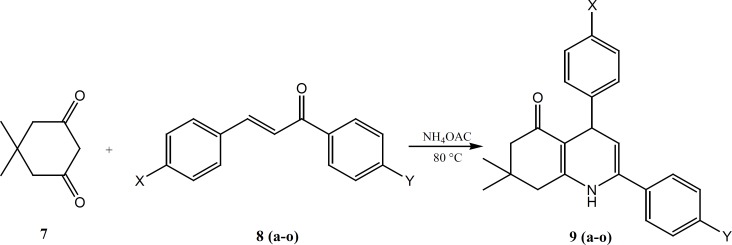
Synthesis of 5-oxo-1,4,5,6,7,8 hexahydroquinoline derivatives 9a-o

The two sets of 5-oxo-1,4,5,6,7,8 hexahydroquinoline regioisomers in which the 4-methanesulfonyl phenyl substituent is attached to C-2 (9a-g) or to C-4 (9h-n), were synthesized in 48-97% yield using a one-pot Hansch reaction as shown in Scheme 1 (8). Accordingly, a mixture of 5, 5-dimethyl-1, 3-cyclohexandione, 1, 3-diaryl-2-propen-1-one and ammonium acetate dissolved in methanol and was refluxed for overnight. The completion of the reaction was monitored by TLC. 

1, 3-Diaryl-2-propen-1-ones (8a-n) were prepared according to our previously literature procedure (4). 

*General procedure for the synthesis of (E)-1, 3-diaryl prop-2-en-1-ones (9a-h)*

A mixture of 5, 5-dimethyl-1,3- cyclohexandion (3 mmol), 1,3-diaryl-2-propen- 1-one (2 mmol), ammonium acetate (4mmol) dissolved in 15 mL methanol and was refluxed at 80 °C for overnight. The completion of the reaction was monitored by TLC. After completion of the reaction, the mixture was cooled to room temperature; ethanol (10 mL) was added to dilute mixture. The mixture was poured into 80 ml ice-water, the precipitate was filtered off and washed with water, and the crude products were obtained. The crude products were purified by recrystallization from ethanol to give final products. 

*7, 8-Dihydro-7,7-dimethyl-2-(4-methylsulfonyl) phenyl)-4-phenylquinolin-5-(1H,4H,6H)-one (9a)*

Yield, 76 %; mp 229-231 °C; IR(KBr disk) υ (cm-1) 1150, 1300 (SO2), 1400-1600 (aromatic), 1667 (C=O), 3254 (NH); 1HNMR (CDCl3, 500 MHz): δ 1.07 (s, 3H, CH3), 1.15 (s, 3H,CH3), 2.21–2.31 (q, 2H, dihydroquinoline H8), 2.39-2.48 (q, 2H, dihydroquinoline H6, *J=*16.2 Hz), 3.08 (s, 3H, SO2Me), 4.79 (d, 1H, dihydroquinoline H4, *J=*5.2 Hz), 5.44 (d, 1H, dihydroquinoline H3, *J=*5.3 Hz), 5.88 (s, 1H, NH), 7.17–7.20 (t, 1H, phenyl H4), 7.29–7.32 (t, 2H, phenyl H3 and H5), 7.38 (d, 2H, phenyl H2 and H6, *J= *7.0 Hz), 7.64(d, 2H, methanesulfonyl phenyl H2 and H6, *J=*8.4 Hz), 7.96 (d, 2H, methanesulfonyl phenyl H3 and H5, *J=*8.4 Hz); Anal. Calcd. for C25H27NO3S: C, 71.23; H, 6.46; N, 3.32. Found: C, 71.46; H, 6.55; N, 3.22.

*7, 8-Dihydro-7, 7-dimethyl-4-(4-methylphenyl)-2-(4-methylsulfonyl) phenyl) quinolin-5-(1H, 4H, 6H)-one (9b)*

Yield, 51 % ; mp 250-253 °C; IR(KBr disk) υ (cm-1) 1150, 1300 (SO2); 1400-1600 (aromatic); 1667 (C=O); 3254 (NH); 1HNMR (CDCl3, 500 MHz): δ 1.08 (s, 3H, CH3), 1.15 (s, 3H, CH3), 2.22–2.47 (m, 4H, dihydroquinoline H6 and H8), 2.32 (s, 3H, CH3), 3.08 (s, 3H, SO2Me), 4.76 (d, 1H, dihydroquinoline H4, *J= *5.1 Hz), 5.44 (d, 1H, dihydroquinoline H3, *J=*5.2 Hz), 5.68 (s, 1H, NH), 7.08-7.12 (m ,2H, *p*-toluoyl H3 and H5) , 7.25–7.27 (m, 2H, *p*-toluoyl H2 and H6), 7.64 (d, 2H, methane sulfonyl phenyl H2 and H6, *J=*8.3 Hz), 7.98 (d, 2H, methanesulfonyl phenyl H3and H5, *J= *8.3 Hz); Anal. Calcd. for C24H25NO3S : C, 70.73; H, 6.18; N, 3.44. Found: C, 70.53; H, 6.32; N, 3.52.

*7, 8-Dihydro-7, 7-dimethyl-4-(4-methoxyphenyl)-2-(4-(methylsulfonyl) phenyl) quinolin-5(1H, 4H, 6H)-one (9c)*

Yield, 56 %; mp 250-253 °C; IR (KBr disk) υ (cm-1) 1150, 1300 (SO2); 1400-1600 (aromatic); 1665 (C=O); 3240 (NH); 1HNMR (CDCl3, 500 MHz): δ 0.98 (s, 3H, CH3), 1.06 (s, 3H, CH3), 2.10–2.13 (t, 1H, dihydroquinoline H8*)*, 2.21 (d, 1H, dihydroquinoline H8, *J=*16.3 Hz), 2.41 (d, 2H, dihydroquinoline H6, *J=*16.4 Hz), 3.01 (s, 3H, SO2Me), 3.7 (s, 3H, OCH3), 4.66 (d, 1H, dihydroquinoline H4, *J= *5.3 Hz), 5.32 (d, 1H , dihydroquinoline H3, *J= *5.3 Hz) , 6.77 (d, 2H, 4-methoxyphenyl H3 and H5, *J= *8.6 Hz), 7.20-7.24 (m, 2H, 4-methoxyphenyl H2 & H6), 7.64 (d, 2H, methanesulfonyl phenyl H2 and H6, *J=*8.1 Hz), 7.88 (d, 2H, methanesulfonyl phenyl H3 and H5, *J=*8.4 Hz); Anal. Calcd. for C25H27NO4S : C, 68.62; H, 6.22; N, 3.20. Found: C, 68.89; H, 6.36; N, 3.39.

*7, 8-Dihydro-4-(4-fluorophenyl)-7, 7-dimethyl-2-(4-methylsulfonyl) phenyl) quinolin-5-(1H, 4H, 6H)-one (9d)*

Yield, 89 % ; mp 130-133 °C; IR (KBr disk) υ (cm-1) 1150, 1300 (SO2); 1400-1600 (aromatic); 1669(C=O); 3390 (NH); 2.2 (d, 1H, dihydroquinoline H8*, J= *16.1 Hz), 2.3-2.37 (m, 2H, dihydroquinoline H6 & H8), 2.45 (d, 1H, dihydroquinoline H6, *J=*16.3 Hz), 3.0 (s, 3H, SO2Me), 4.88 (d, 1H, dihydroquinoline H4, *J= *5.0 Hz), 5.1 (d, 1H, dihydroquinoline H3, *J= *5.0 Hz), 5.77 (s, 1H, NH), 7.10-7.22 (t, 2H, 4-fluorophenyl H3 and H5), 7.40-7.42 (q, 2H, 4-fluorophenyl H2 and H6), 7.58 (d, 2H, methanesulfonyl phenyl H2 & H6, *J*=8.8 Hz), 7.9 (d, 2H, methanesulfonyl phenyl H3 and H5, *J=*8.2 Hz); Anal. Calcd. for C24H24FNO3S: C, 67.74; H, 5.67; N, 3.29. Found**: **C, 67.94; H, 5.81; N, 3.12.

*4-(4-Chlorophenyl)-7, 8-dihydro-7, 7-dimethyl-2-(4-methylsulfonyl) phenyl) quinolin-5-(1H, 4H, 6H)-one (9e)*

Yield, 86 %; mp 232-236 °C; IR (KBr disk) υ (cm-1) 1150, 1300 (SO2); 1400-1600 (aromatic); 1669(C=O); 3248 (NH); 1HNMR (CDCl3, 500 MHz): δ 1.07 (s, 3H, CH3), 1.15 (s, 3H, CH3), 2.21–2.31 (q, 2H, dihydroquinoline H8), 2.33-2.47 (q, 2H, dihydroquinoline H6 , *J=*16.2 Hz), 3.08 (s, 3H, SO2Me) , 4.75 (d, 1H, dihydroquinoline H4, *J=*5.3 Hz), 5.44 (d, 1H, dihydroquinoline H3, *J=*5.3 Hz), 5.78 (s, 1H, NH), 6.85 (d, 2H, 4-cholorophenyl H3 and H5, *J= *9.6 Hz),7.29 (m, 2H, 4-cholorophenyl H2 & H6) 7.65 (d, 2H, methanesulfonyl phenyl H2 and H6, *J=*8.4 Hz), 7.98 (d, 2H, methanesulfonyl phenyl H3 and H5, *J=*8.5 Hz); Anal. Calcd. for C24H24ClNO3S : C, 65.22; H, 5.47; N, 3.17. Found: C, 65.54; H, 5.56; N, 3.42.

*4-(4-Bromophenyl)-7, 8-dihydro-7, 7-dimethyl-2-(4-methylsulfonyl) phenyl) quinolin-5-(1H, 4H, 6H)-one (9f)*

Yield, 88 %; mp 237-240 °C; IR (KBr disk) υ (cm-1) 1150, 1300 (SO2); 1400-1600 (aromatic); 1661(C=O); 3198 (NH); 1HNMR (CDCl3, 500 MHz): δ 0.98 (s, 3H, CH3), 1.07 (s, 3H, CH3), 2.14–2.19 (m, 2H, dihydroquinoline H8), 2.2-2.1 (q, 2H, dihydroquinoline H6 ), 3.02 (s, 3H, SO2Me), 4.18-4.21 (t, 1H, dihydroquinoline H4), 4.69 (d, 1H, dihydroquinoline H3, *J=*5.3 Hz), 5.27 (d, 1H, NH), 7.17 (d, 2H, 4-bromophenyl H3 and H5, *J= *8.3 Hz), 7.32 (d, 2H, 4-bromophenyl H2 & H6, *J= *8.3 Hz), 7.64 (d, 2H, methanesulfonyl phenyl H2 and H6, *J=*8.3 Hz), 7.90 (d, 2H, methanesulfonyl phenyl H3 and H5, *J=*8.4 Hz); Anal. Calcd. for C24H24BrNO3S : C, 59.29; H, 4.97; N, 2.88. Found: C, 59.60; H, 5.11; N, 3.02.

*7, 8-Dihydro-7, 7-dimethyl-2-(4-methylsulfonyl) phenyl)-4-(4-nitrophenyl) quinolin-5-(1H, 4H, 6H)-one (9g)*

Yield, 97 %; mp 234-240 °C ; IR (KBr disk) υ (cm-1) 1150, 1300 (SO2); 1400-1600 (aromatic); 1661(C=O); 3238 (NH); 1HNMR (CDCl3, 500 MHz): δ 0.98 (s, 3H, CH3), 1.08 (s, 3H, CH3), 2.11–2.24 (m, 2H, dihydroquinoline H8*) *, 2.37-2.45 (q, 2H, dihydroquinoline H6 ), 3.03 (s, 3H , SO2Me), 4.86 (d, 1H, dihydroquinoline H4, *J= *5.1 Hz) , 5.24 (d, 1H, dihydroquinoline H3, *J= *5.1 Hz), 7.4 (d, 2H, 4-nitrophenyl H2 and H6, *J= *8.6 Hz), 7.66 (d, 2H, 4-nitrophenyl H2 & H6, *J= *8.4 Hz), 7.92 (d, 2H, methanesulfonyl phenyl H2 and H6, *J=*8.4 Hz), 8.10 (d, 2H, methanesulfonyl phenyl H3 and H5, *J=*8.5 Hz); Anal. Calcd. for C24H24N2OS5: C, 63.70; H, 5.35; N, 6.19. Found: C, 63.81; H, 5.61; N, 6.43.

*7, 8-Dihydro-7, 7-dimethyl-4-(4-methylsulfonyl) phenyl)-2-phenylquinolin-5 (1H, 4H, 6H)-one (9h)*

Yield, 78 %; mp 205-208 °C; IR (KBr disk) υ (cm-1) 1150, 1300 (SO2); 1400-1600 (aromatic); 1667 (C=O); 3356 (NH); 1HNMR (CDCl3): δ 1.07 (s, 3H, CH3), 1.16 (s, 3H, CH3), 2.23 (d, 1H, dihydroquinoline H8*, J= *16.5 Hz), 2.32 (d, 1H, dihydroquinoline H8*, J=*16.5 Hz), 2.38 (d, 1H, dihydroquinoline H6, *J=*16.3 Hz), 2.49 (d, 1H, dihydroquinoline H6, *J= *16.3 Hz), 3.05 (s, 3H, SO2Me), 4.90 (d, 1H, dihydroquinoline H4, *J= *5.0 Hz), 5.25 (d, 1H, dihydroquinoline H3, *J= *5.0 Hz), 5.93 (s, 1H, NH), 7.41-7.48 (m, 5H, phenyl), 7.59 (d, 2H, methanesulfonyl phenyl H2 and H6, *J *= 8.7 Hz), 7.87 (d, 2H, methanesulfonyl phenyl H3 and H5, *J *= 8.7 Hz); Anal. Calcd. for C24H25NO3S : C, 70.73; H, 6.18; N, 3.44. Found: C, 71.03; H, 6.38; N, 3.59.

*7, 8-Dihydro-7, 7-dimethyl-2-(4-methylphenyl)-4-(4-(methylsulfonyl) phenyl) quinolin-5(1H, 4H, 6H)-one (9i)*

Yield, 48%; mp: 223-225 °C; IR (KBr disk) υ (cm-1) 1150, 1300 (SO2); 1400-1600 (aromatic); 1685(C=O); 3024 (NH) ;1HNMR (CDCl3, 500 MHz): δ 1.05 (s, 3H, CH3), 1.15 (s, 3H, CH3), 2.20 (d, 1H, dihydroquinoline H8*, J=*16.1 Hz), 2.29-2.37 (m, 2H, dihydroquinoline H6 and H8), 2.40 (s, 3H, CH3), 2.45-2.48 (d, 1H, dihydroquinoline H6, *J= *16.2 Hz), 3.03 (s, 3H, SO2Me), 4.88 (d, 1H, dihydroquinoline H4, *J= *5.0 Hz), 5.21 (d, 1H, dihydroquinoline H3, *J= *5.0 Hz), 5.88 (s, 1H, NH), 7.32 (d, 2H, *p*-toluoyl H3 and H5*, J =*8.0 Hz), 7.58 (d, 2H*, p*-toluoyl H2 and H6*, J=*8.3 Hz), 7.57 (d, 2H, methanesulfonyl phenyl H2 and H6*, J =*8.3 Hz), 7.86 (d, 2H, methanesulfonyl phenyl H3 and H5, 

*J =*8.3 Hz); Anal. Calcd. for C25H27NO3S : C, 71.23; H, 6.46; N, 3.32. Found: C, 71.54; H, 6.67; N, 3.39.

*7, 8-Dihydro-2-(4-methoxyphenyl)-7, 7-dimethyl-4-(4-(methylsulfonyl) phenyl) quinolin-5(1H, 4H, 6H)-one (9j)*

Yield, 53 %; mp, 226-230 °C; IR (KBr disk) υ (cm-1) 1150, 1300 (SO2); 1400-1600 (aromatic); 1664 (C=O); 3342 (NH); 1HNMR (CDCl3): δ 1.05 (s, 3H, CH3), 1.15 (s, 3H, CH3), 2.21 (d, 1H, dihydroquinoline H8*, J=*16.3 Hz), 2.33 (m, 2H, dihydroquinoline H6 and H8), 2.46 (d, 1H, dihydroquinoline H6, *J=*16.3 Hz), 2.49 (d, 1H, dihydroquinoline H6, *J=*16.3 Hz), 3.03 (s, 3H, SO2Me), 3.85 (s, 3H, OCH3), 4.87 (d, 1H, dihydroquinoline H4 , *J= *5.0 Hz), 5.15 (d, 1H, dihydroquinoline H3, *J= *5.0 Hz), 5.88 (s, 1H, NH), 6.94 (d, 2H, 4**-**methoxyphenyl H3 & H5*, J=*8.7 Hz), 7.36 (d, 2H, 4-methoxyphenyl H2 and H6, *J=*8.7 Hz), 7.58 (d, 2H, methanesulfonyl phenyl H2 and H6 , *J *=8.2 Hz ), 7.86 (d, 2H, methanesulfonyl phenyl H3 & H5, *J *=8.2 Hz); Anal. Calcd. for C25H27NO4S : C, 68.62; H, 6.22; N, 3.20. Found: C, 68.74; H, 5.99; N, 3.31.

*2-(4-Fluorophenyl)-7, 8-dihydro-7, 7-dimethyl-4-(4-(methylsulfonyl) phenyl) quinolin-5 (1H, 4H, 6H)-one (9k)*

Yield, 89 %; mp, 226-230 °C; IR (KBr disk) υ (cm-1) 1150, 1300 (SO2); 1400-1600 (aromatic); 1664 (C=O); 3028 (NH); 1HNMR (CDCl3): δ 1.06 (s, 3H, CH3), 1.16 (s, 3H,CH3), 2.22 (d, 1H, dihydroquinoline H8*, J= *16.3 Hz), 2.29-2.37 (m, 2H, dihydroquinoline H6 and H8), 2.47 (d, 1H, dihydroquinoline H6, *J=*16.3 Hz), 3.04 (s, 3H, SO2Me), 4.88 (d, 1H, dihydroquinoline H4, *J= *5.0 Hz), 5.18 (d, 1H, dihydroquinoline H3, *J= *5.0 Hz), 5.77 (s, 1H, NH), 7.10-7.13 (t, 2H, 4-fluorophenyl H3 and H5), 7.40-7.42 (q, 2H, 4-fluorophenyl H2 and H6), 7.58 (d, 2H, methanesulfonyl phenyl H2 and H6, *J*=8.8 Hz), 7.87 (d, 2H, methanesulfonyl phenyl H3 and H5, *J=*8.2 Hz); Anal. Calcd. for C24H24FNO3S : C, 67.74; H, 5.68; N, 3.29. Found: C, 67.88; H, 5.75; N, 3.46.

*2-(4-Cholorophenyl)-7, 8-dihydro-7, 7-dimethyl-4-(4-(methylsulfonyl) phenyl) quinolin-5(1H, 4H, 6H)-one (9i)*

Yield, 82 %; mp 226-230 °C; IR (KBr disk) υ (cm-1) 1150, 1300 (SO2); 1400-1600 (aromatic); 1654 (C=O); 3342 (NH); 1HNMR (CDCl3): δ 1.07 (s, 3H, CH3), 1.17 (s, 3H, CH3), 2.24 (d, 1H, dihydroquinoline H8*, J=*16.4 Hz), 2.30-2.35 (m, 2H, dihydroquinoline H6 and H8), 2.50 (d, 1H, dihydroquinoline H6, *J=*16.3 Hz), 3.05 (s, 3H, SO2Me), 4.88 (d, 1H, dihydroquinoline H4, *J= *5.0 Hz), 5.23 (d, 1H, dihydroquinoline H3, *J= *5.0 Hz), 5.84 (s, 1H, NH), 7.37-7.38 (m, 4H, 4-chlorophenyl), 7.58 (d, 2H, methanesulfonyl phenyl H2 and H6, *J *=8.2 Hz), 7.88 (d, 2H, methanesulfonyl phenyl H3 and H5, *J *=8.2 Hz) ); Anal. Calcd. for C24H24ClNO3S: C, 65.22; H, 5.47; N, 3.17. Found: C, 65.36; H, 5.69; N, 3.32

*2-(4-Bromophenyl)-7, 8-dihydro-7, 7-dimethyl-4-(4-(methylsulfonyl) phenyl) quinolin-5 (1H, 4H, 6H)-one (9m)*

Yield, 87 %; mp 226-230 °C; IR (KBr disk) υ (cm-1) 1150, 1300 (SO2); 1400-1600 (aromatic); 1664 (C=O); 3355 (NH); 1HNMR (CDCl3): δ 1.06 (s, 3H, CH3), 1.16 (s, 3H, CH3), 2.23 (d, 1H, dihydroquinoline H8*, J=*16.4 Hz), 2.29-2.38 (m, 2H, dihydroquinoline H6 and H8), 2.47 (d, 1H, hydroquinoline H6, *J=*16.4 Hz), 3.04 (s, 3H, SO2Me), 4.87 (d, 1H, dihydroquinoline H4, *J= *5.1 Hz), 5.23 (d, 1H, dihydroquinoline H3, *J= *4.9 Hz), 5.81 (s, 1H, NH), 7.31 (d, 2H, methanesulfonyl phenyl H2 and H6, *J*=8.8 Hz), 7.54–7.57 (m, 4H, 4-bromophenyl), 7.87 (d, 2H, methanesulfonyl phenyl H3 and H5, *J*=8.2 Hz); Anal. Calcd. for C24H24BrNO3S: C, 59.26; H, 4.97; N, 2.88. Found: C, 59.39; H, 5.12; N, 3.01.

*7, 8-Dihydro-7, 7-dimethyl-4-(4-(methylsulfonyl) phenyl)-2-(4-nitrophenyl) quinolin-5 (1H, 4H, 6H)-one (9n)*

Yield, 93 %; mp 226-230 °C; IR (KBr disk) υ (cm-1) 1150, 1300 (SO2); 1400-1600 (aromatic); 1664 (C=O); 3300 (NH); 1HNMR (CDCl3, 500 MHz): δ 1.07 (s, 3H, CH3), 1.17 (s, 3H, CH3), 2.20–2.24 (t, 1H, dihydroquinoline H8) , 2.33 (d, 1H, dihydroquinoline H8 , *J= *16.3 Hz), 2.42 (d, 1H, dihydroquinoline H6, *J=*16.4 Hz), 2.52 ( d, 1H, dihydroquinoline H6*, J=*16.4 Hz), 3.05 (s, 3H, SO2Me) , 4.91 (d, 1H, dihydroquinoline H4, *J= *5.1Hz), 5.39 (d, 1H , dihydroquinoline H3, *J=*5.0 Hz), 5.98 (s, 1H, NH) , 7.57 (d , 2H, 4-nitrophenyl H2 and H6, *J=*8.3 Hz) , 7.61 (d, 2H, 4-nitrophenyl H3 and H5, *J=*8.8 Hz) , 7.87 (d, 2H, methanesulfonyl phenyl H2 and H6, *J=*8.3 Hz), 8.28 (d, 2H, methane sulfonyl phenyl H3 and H5, *J=*8.81 Hz); Anal. Calcd. for C24H24N2O5S: C, 63.70; H, 5.35; N, 6.19. Found: C, 63.94; H, 5.57; N, 6.41.

*Molecular modeling and biological evaluation*

Docking studies were performed using Autodock software Version 3.0. The coordinates of the X-ray crystal structure of the selective COX-2 inhibitor SC-558 bound to the murine COX-2 enzyme was obtained from the RCSB Protein Data Bank (1cx2) and hydrogens were added. The ligand molecules were constructed using the Builder module and were energy minimized for 1000 iterations reaching a convergence of 0.01 kcal/mol Å. The energy minimized ligands were superimposed on SC-558 in the PDB file 1cx2 after which SC-558 was deleted. The aim of docking is to search for suitable binding configuration between the ligands and the rigid protein. These docked structures were very similar to the minimized structures provided initially. The quality of the docked structures was determined by measuring the intermolcular energy of the ligand-enzyme assembly (9).

*In-vitro cyclooxygenase (COX) inhibition assays*

The assay was performed using an enzyme chemiluminescent kit (Cayman chemical, MI, USA) according to our previously reported method (10).

## Results and Discussion

**Figure 2:**
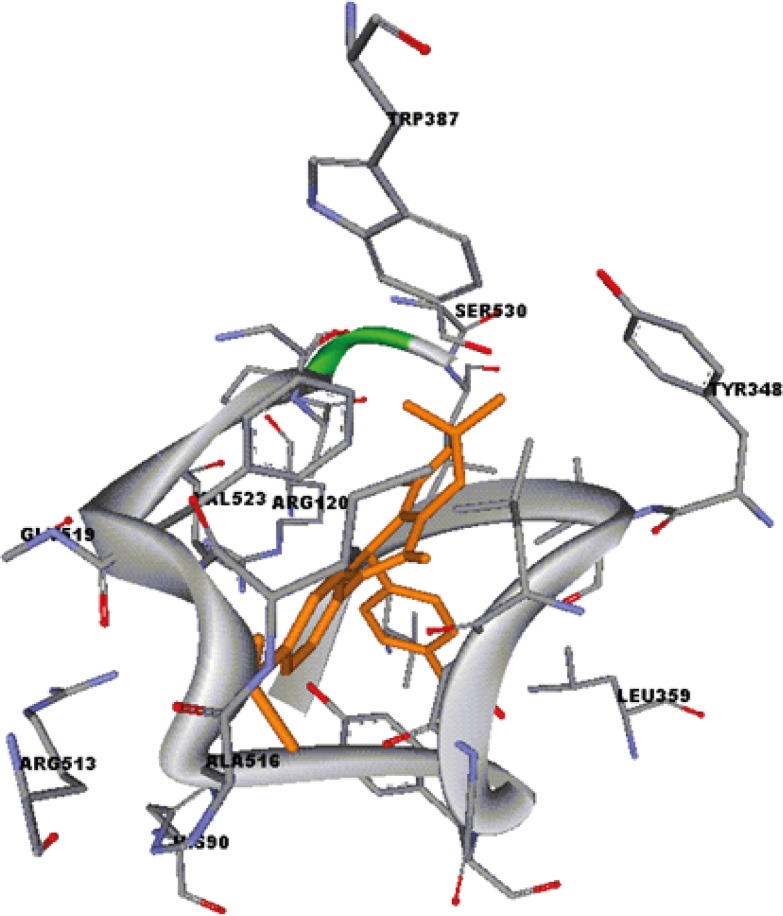
7, 8-Dihydro-2-(4-methoxyphenyl)-7,7-dimethyl-4-(4-(methylsulfonyl)phenyl)quinolin-5(1*H*, 4*H*,6*H*)-one (9c) (orange) docked in the active site of murine COX-2. Hydrogen atoms of the amino acid residues have been removed to improve clarity

A group of 5-oxo-1,4,5,6,7,8 hexahydroquinolines possessing a MeSO2 group at the *para-*position of the C-2 phenyl ring containing different substituents (4-F, 4-Cl, 4-Br, 4-OMe, 4-Me, 4-NO2) at the *para-*position of the C-4 phenyl ring (9a-g), and the corresponding regioisomers (9h-n), were prepared to study the effect of these substituents on COX-2 selectivity and potency. SAR data (IC50 M values) obtained by determination of the in vitro ability of the synthesized compounds to inhibit the COX-1 and COX-2 isozymes showed that the position of the COX-2 SO2Me pharmacophore and the nature of the *para-*substituents on the C-2 or C-4 phenyl ring were important on COX-2 inhibitory potency and selectivity. In vitro COX-1/COX-2 inhibition studies showed that compounds having a MeSO2 group at the *para*-position of the C-2 phenyl ring (9a-g) were more selective COX- 2 inhibitors compared to their corresponding regioisomers (9h-n). These results also indicated that incorporation of a methoxy (OMe) substituent at the *para-*position of the C-2 or C-4 phenyl ring increased the potency and COX-2 selectivity. Accordingly, compounds 9c and 9j showed the best activity among the synthesized compounds (9c, IC50 = 0.17 M, S.I. = 3.3; 9j, IC50 = 0.30 M, S.I. = 62.3). In contrast introduction of large groups such as Cl, Br or NO2 at the same position of C-2 phenyl (9e-g) and C-4 phenyl (9l-n) decreased COX-2 inhibitory potency and selectivity. However, the two regioisomers having an unsubstituted C-2 phenyl (9a), or C-4 phenyl (9h), ring were approximately equipotent inhibitors of COX-2 and showed similar selectivity. Our results indicated that 7, 8-dihydro-7,7-dimethyl-2-(4-methoxyphenyl)- 4-(4-(methylsulfonyl)phenyl)quinolin-5(1*H*,4*H*, 6*H*)-one (9c), showed the optimal combination of COX-2 inhibitory potency and selectivity. A molecular modeling study of the most selective COX-2 inhibitor compound 9c docked in the COX-2 active site (Figure 2) shows that it binds in the primary binding-site such that the *p*-SO2Me substituent on the C-2 phenyl ring is well oriented into secondary pocket present in COX-2. One of the *O*-atoms of the SO2Me moiety forms a *H*-bond with the N*H2 *of Arg513 (distance = 3.1 Å), whereas the other *O*-atom is closer to the N*H *of His90 (distance = 3.0 Å). In addition, the N-*H *of the central ring is near to C=*O *of Val349and can form hydrogen bonding interaction with this amino acid. (Distance = 3.9 Å). Moreover, the carbonyl group of 5-oxo-1, 4, 5,6,7,8 hexahydroquinolines is close to N*H *of Arg120 (distance < 3Å) and can form *H*-bond with the N*H *of Arg120. These observations together with experimental results provide a good explanation for the potent and selective inhibitory activity exhibited by 9c.

**Table 1:** *In-vitro* COX-1 and COX-2 enzyme inhibition data for compounds 9a-o

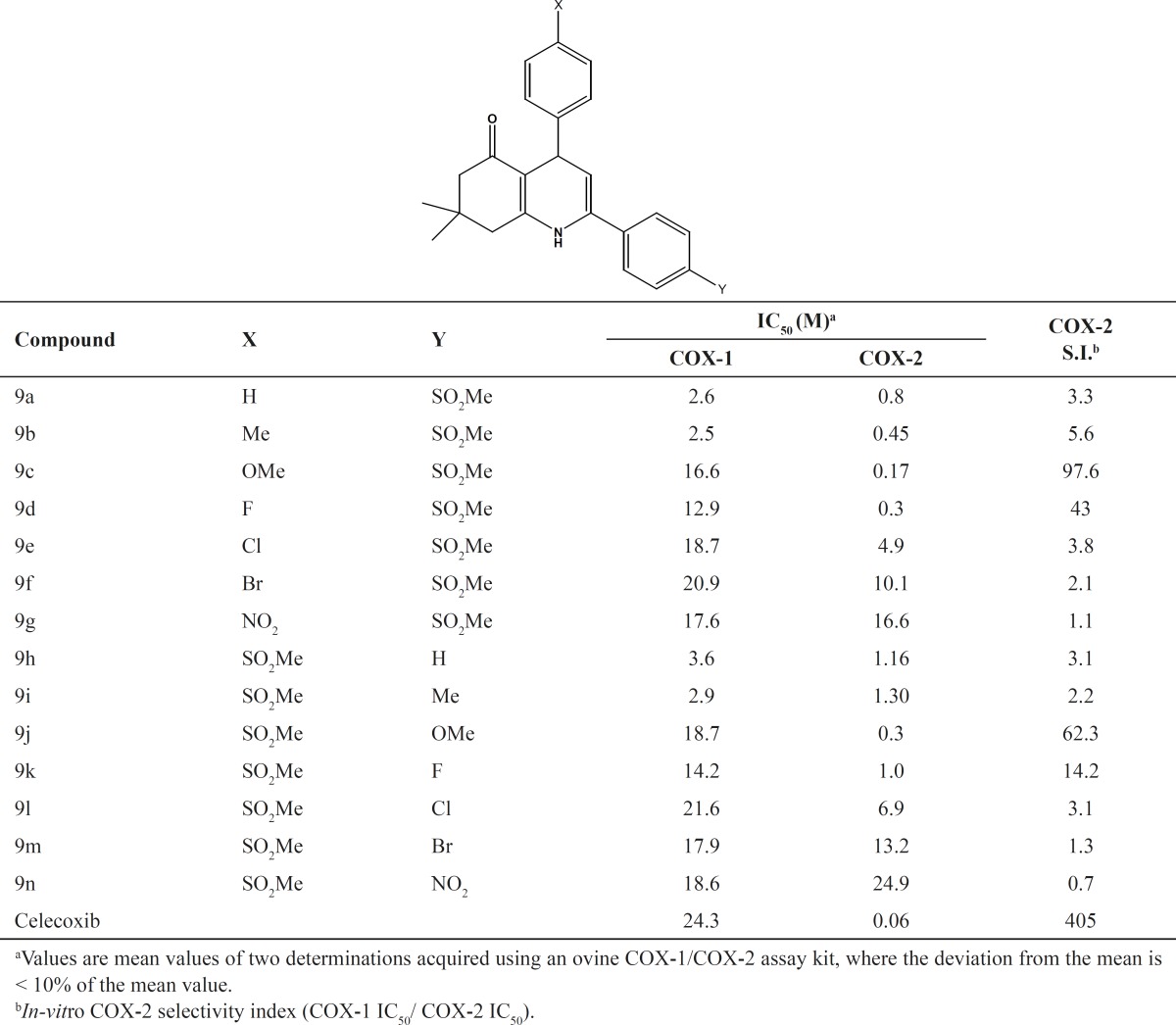

^a^Values are mean values of two determinations acquired using an ovine COX-1/COX-2 assay kit, where the deviation from the mean is < 10% of the mean value.

^b^ *In-vit*ro COX-2 selectivity index (COX-1 IC_50_/ COX-2 IC_50_).

## Conclusions

A new class of 5-oxo-1, 4, 5,6,7,8 hexahydroquinolines that are readily accessible via a simple Hansch reaction, was designed for evaluation as COX-2 inhibitors. In vitro enzyme inhibition structure-activity studies indicated that (i) the hexahydroquinoline moiety present in a 2,4-diaryl-5-oxo-1,4,5,6,7,8 hexahydroquinoline structure is a suitable scaffold (template) to design COX-2 inhibitors, and (ii) 7,8-dihydro-7,7-dimethyl-2-(4-methoxyphenyl)-4-(4-(methylsulfonyl)phenyl)quinolin-5(1*H*,4*H*,6*H*)-one (9c) is not only a potent, but also a selective COX-2 inhibitor.
